# Cognitive dysfunction, elevated anxiety, and reduced cocaine response in circadian clock-deficient cryptochrome knockout mice

**DOI:** 10.3389/fnbeh.2013.00152

**Published:** 2013-10-24

**Authors:** Dimitri De Bundel, Giuseppe Gangarossa, Anne Biever, Xavier Bonnefont, Emmanuel Valjent

**Affiliations:** ^1^CNRS, UMR-5203, Institut de Génomique FonctionnelleMontpellier, France; ^2^INSERM, U661Montpellier, France; ^3^Universités de Montpellier 1 and 2, UMR-5203Montpellier, France

**Keywords:** Cry1 and Cry2 knockout mice, mood-related disorder, psychostimulants, learning

## Abstract

The circadian clock comprises a set of genes involved in cell-autonomous transcriptional feedback loops that orchestrate the expression of a range of downstream genes, driving circadian patterns of behavior. Cognitive dysfunction, mood disorders, anxiety disorders, and substance abuse disorders have been associated with disruptions in circadian rhythm and circadian clock genes, but the causal relationship of these associations is still poorly understood. In the present study, we investigate the effect of genetic disruption of the circadian clock, through deletion of both paralogs of the core gene *cryptochrome* (*Cry1* and *Cry2*). Mice lacking *Cry1* and *Cry2* (*Cry1^−/−^Cry2^−/−^*) displayed attenuated dark phase and novelty-induced locomotor activity. Moreover, they showed impaired recognition memory but intact fear memory. Depression-related behaviors in the forced swim test or sucrose preference tests were unaffected but *Cry1^−/−^Cry2^−/−^* mice displayed increased anxiety in the open field and elevated plus maze tests. Finally, hyperlocomotion and striatal phosphorylation of extracellular signal-regulated kinase (ERK) induced by a single cocaine administration are strongly reduced in *Cry1^−/−^Cry2^−/−^* mice. Interestingly, only some behavioral measures were affected in mice lacking either *Cry1* or *Cry2*. Notably, recognition memory was impaired in both *Cry1^−/−^Cry2^+/+^* and *Cry1^+/+^Cry2^−/−^* mice. Moreover, we further observed elevated anxiety in *Cry1^−/−^Cry2^+/+^* and *Cry1^+/+^Cry2^−/−^* mice. Our data indicate that beyond their role in the control of circadian rhythm, *cryptochrome* genes have a direct influence in cognitive function, anxiety-related behaviors and sensitivity to psychostimulant drugs.

## Introduction

In mammals, light entrains the central circadian oscillator in the suprachiasmatic nucleus of the hypothalamus (SNC), which in turn synchronizes subsidiary oscillators throughout the brain and other organs. This hierarchical system controls circadian activity and orchestrates the expression of a range of downstream genes that allow the organism to anticipate daily changes in the environment (Mohawk et al., 2012). These events are tightly regulated by the molecular clockwork, which is composed of a series of genes involved in transcriptional feedback loops with a circadian oscillation of activity (Mohawk et al., 2012). The translation products of the circadian clock genes *Circadian locomotor output cycles kaput* (CLOCK) and *PAS-domain protein 2* (NPAS2) form heterodimers with brain and muscle *ARNTL*-like proteins (BMAL1, BMAL2) to activate the transcription of other genes, including *periods* (*mPer1*, *mPer2*, *mPer3*) and *cryptochromes* (*Cry1*, *Cry2*). Their translation products, PER and CRY, in turn heterodimerize and repress the transcriptional activity of the complex formed by CLOCK/NPAS2-BMAL1/2 (Takahashi et al., 2008). As such, clock genes control circadian activity allowing the organism to anticipate daily changes in the environment (Takahashi et al., 2008; Mohawk et al., 2012).

Beyond their role in the control of circadian rhythm by light, several studies have pointed out that circadian clock genes may have a more widespread influence on cognition, mood, anxiety, and reward-related behaviors (Wulff et al., 2010). Single nucleotide polymorphisms in core circadian clock genes have been associated with autism spectrum disorders (Nicholas et al., 2007); attention deficit hyperactivity disorder (Kissling et al., 2008; Xu et al., 2010), major depressive disorder (Partonen et al., 2007; Lavebratt et al., 2010; Soria et al., 2010), bipolar disorder (Nievergelt et al., 2006; Shi et al., 2008; Soria et al., 2010), anxiety disorders (Sipila et al., 2010) and substance abuse disorders (Kovanen et al., 2010; Dong et al., 2011; Wang et al., 2012). However, the causal relationship for these associations remains poorly understood. Circadian clock genes may affect specific aspects of psychiatric disorders through circadian control or through distinct regulation of downstream effectors. Indeed, an emerging body of evidence suggests opposing actions on behavioral output by circadian transcriptional activators (CLOCK, NPAS2, BMAL1, BMAL2) and transcriptional repressors (PER1, PER2, CRY1, CRY2). Moreover, circadian clock genes exist in paralog pairs with often complementary but not necessarily identical functions (Vitaterna et al., 1999; Zheng et al., 2001; De Bundel et al., 2013; Shi et al., 2010). These functional differences may reflect region-dependent clock gene expression and paralog-specific control over output genes (Hampp and Albrecht, 2008).

In the present study, we perform an in depth characterization of behaviors relevant to psychiatric disease in *Cry1*^−/−^/*Cry2*^+/+^, *Cry1*^+/+^/*Cry2*^−/−^, and *Cry1*^−/−^/*Cry2*^−/−^ mice. In doing so, we aim to address the causality between circadian gene disruption and psychiatric disease. These novel findings reveal previously unappreciated and complementary roles for Cry1 and Cry2 in regulating cognitive function, anxiety-like behavior and sensitivity to psychostimulants and support the hypothesis that circadian activator and repressor genes exert opposite control over a range of behavioral outputs.

## Materials and methods

### Animals and ethics

Double heterozygous *Cry1^+/−^/Cry2^+/−^* mice were obtained by breeding *Cry1^+/−^* and *Cry2^+/−^* mice (van der Horst et al., 1999), previously backcrossed for at least 12 generations with the C57/Bl6 strain (Charles River, L'Arbresle, France). They were then intercrossed to generate *Cry1^−/−^/Cry2^+/+^*, *Cry1^+/+^/Cry2^−/−^*, *Cry1^−/−^/Cry2^−/−^* mice and their control wild type (WT) littermates. Initial experiments were carried out on a cohort of WT and *Cry1^−/−^/Cry2^−/−^* mice. Further comparisons between all genotypes were performed on a separate cohort of animals and are represented in the table and separate graphs. All animals were housed in a 12 h-light/12 h-dark light cycle with food and water provided *ad libitum*. Animals were housed under standard laboratory conditions with free access to food and water in a 12 h light-dark cycle with lights on at 7:00 (ZT 0). All behavioral experiments were carried out during the light phase between ZT 4–10 with male mice, since baseline activity showed minimal differences between genotypes at this time of the light-dark cycle. Mice were age-matched, between 8 and 12 weeks. Spontaneous locomotor activity, open field activity, objects recognition memory and anxiety in the elevated plus maze were determined on the same animals in this order in a series of behavioral tests. Next, one of the following behaviors was evaluated for a given batch of mice: sucrose preference, fear conditioning, or cocaine sensitization. All experiments were in accordance with the guidelines of the French Agriculture and Forestry Ministry for handling animals (authorization number/license D34-172-13).

### Novelty-induced and circadian locomotor activity

Horizontal and vertical activity was measured in a circular corridor (Imetronic, Pessac, France). Counts for horizontal activity were incremented by consecutive interruption of two adjacent beams placed at a height of 1 cm per 90° sector of the corridor (mice moving through one-quarter of the circular corridor) and counts for vertical activity (rearings) corresponding to interruption of beams placed at a height of 7.5 cm along the corridor (mice stretching upwards) were used as an additional measure for exploratory activity. Data were pooled for all experiments during which mice were habituated to the circular corridor. For circadian locomotor activity, a 12 h dark/light cycle was maintained. Naïve animals were introduced into the apparatus at ZT 5 and had free access to food and water for the duration of the experiment. ZT5 was selected as the start time of the experiment to enable habituation to the apparatus prior to the onset of the dark phase, as described previously (McClung et al., 2005).

### Object recognition

Mice were habituated to an open field (white plastic box with 35 cm width × 45 cm length × 25 cm height) for 10 min on 2 consecutive days. The following day, mice were allowed to freely explore 2 objects (A and B) placed 10 cm from opposite walls of the open field until the criterion of 40 s total object interaction was reached. Object interaction was defined as approaching the object with the nose closer than 1 cm. Following a retention interval of 24 h, mice underwent a 5 min recall session during which the open field contained a familiar object (A) and a novel object (C). Replicate objects were used to avoid smell traces. Following each session, the objects and the open field were cleaned with 70% ethanol. The experiments were filmed and an observer blinded to genotype performed scoring. A retention index was calculated using the formula RI = (C−A)/(A+C).

### Fear conditioning

Pavlovian fear conditioning was performed in a conditioning box (20 cm width × 20 cm length × 20 cm height) placed within a sound proof chamber (Panlab, Harvard Apparatus). Two different contexts were used for training and testing (A: white walls, metal grid on black floor, washed with 1% acetic acid; B: black walls, white rubber floor, washed with 70% ethanol). Mice were conditioned in context A. After 2 min habituation, mice received three pairings (60–120 s variable pairing interval) of a conditioned stimulus (CS: 7.5 kHz, 80 dB, 30 s tone) with an unconditioned stimulus (US: 2 s, 0.6 mA scrambled footshock) using a Freezing system (Panlab, Barcelona, Spain). Following a 24 h retention interval, mice were habituated for 2 min in context B and tested for fear memory by 4 presentations of CS (with 60–120 s variable interval). Freezing was measured using a load cell coupler (Panlab, Barcelona, Spain) and was defined as the lack of activity above a body weight-corrected threshold for a duration of 1 s or more as analyzed using Freezing software (Panlab, Barcelona, Spain).

### Forced swim test

Mice were assessed for spontaneous depression-related behavior using a modified version of the forced swim test (Porsolt et al., 1977). Behavioral despair was evaluated in a single 10 min forced swim session. Mice were placed for 10 min in a glass beaker (15 cm width × 20 cm height) half filled water at 25°C so that they were unable to touch the bottom of the beaker with their hind paws. The beakers were filled with fresh water between sessions. Passive immobility was scored per 2 min intervals by an observer blinded to genotype. Immobility was defined as the lack of movements except those required for keeping the head above the water (essentially no movement in three or four paws).

### Sucrose preference

Sucrose preference was evaluated using a two-bottle free choice paradigm. Mice were housed individually with access to 2 identical drinking bottles containing 1% sucrose in water or water. On 3 consecutive days, liquid consumption in each bottle was measured at the onset of every dark and light phase, while switching the location of the drinking bottles every 12 h to avoid side bias.

### Open field

Spontaneous exploratory behavior was monitored in an open field (white plastic box with 35 cm width × 45 cm length × 25 cm height) for 10 min. The open field was wiped with 70% ethanol between sessions. The center zone was defined as a virtual perimeter within 5 cm from the sides of the box. Experiments were filmed and an observer blinded to genotype scored the time spent in the center (4 paws inside the center zone) and the number of midline crosses (4 paws crossing a midline of the box).

### Elevated plus maze

The elevated plus maze was elevated 1 m above the floor and was constructed of black plastic with 2 open arms (5 cm width × 35 cm length × 0.5 cm height of the walls) and 2 closed arms (5 width × 35 cm length × 15 cm height of the walls). Mice were placed in the center of an elevated plus maze facing one of the closed arms and were allowed to explore the maze for 5 min. The elevated plus maze was wiped with 70% ethanol between sessions. Experiments were filmed and an observer blinded to genotype performed scoring for entries and time spent in the closed arms (4 paws within closed arm) or open arms (4 paws within open arms).

### Rotarod

Motor learning was assessed using a mouse accelerating rotarod (Ugo Basile). Mice were placed on the rotating drum that accelerated from 4 to 40 rpm over 5 min for three trials a day, for 3 consecutive days. The intertrial interval was 45 min for all the mice. Rotarod scores were scored for latency to either fall or ride the rod around.

### Cocaine-induced locomotor activity and sensitization

Cocaine hydrocloride (Sigma) was dissolved in 0.9% physiological saline and injected intraperitoneally (i.p.) in a body volume of 10 ml/kg. Locomotor activity was measured in the same apparatus as used for novelty-induced and circadian locomotor activity, as described previously (Brami-Cherrier et al., 2005). All mice were habituated to the test apparatus, handling, and procedure for three consecutive days before the actual experiment. In this habituation procedure mice were placed for 30 min in the activity box, received a first injection of saline, and were placed back in the box for 1 h. For the acute drug injection (day 4), the handling was identical, except that the saline injection was replaced by cocaine injection (7.5 and 15 mg/kg, i.p.) as described previously (Bertran-Gonzalez et al., 2008). In cocaine sensitization experiments, mice were treated repeatedly with cocaine (7.5 and 15 mg/kg, i.p., every day) for 5 consecutive days. This repeated exposure was followed by 12 days of withdrawal and by a challenge injection of cocaine (7.5 and 15 mg/kg). Locomotor activity was measured as described above on day 4.

### Immunoblotting

Mice were sacrificed for preparation of the samples for western blotting between ZT 4–6. After immersion in liquid nitrogen for 4 s, the brains were removed and the striata were quickly dissected out on ice-cold surface, sonicated in 350 μl of 10% SDS and boiled for 10 min. Aliquots (2.5 μl) of the homogenate were used for protein determination using a BCA assay kit (Pierce Europe). Equal amounts of proteins (15 μg) for each sample were loaded onto 8–10% polyacrylamide gels. Proteins were separated by SDS-PAGE and transferred to PVDF membranes (GE Healthcare). The membranes were immunoblotted using anti-diphospho ERK1/2 (1:2000; Cell Signaling Technology), ERK1/2 (1:2000; Cell Signaling Technology) and β-actin (1:40000; Abcam) antibodies. Bound antibodies were detected with HRP-conjugated anti-rabbit or anti-mouse antibodies (1:10000; Cell Signaling Technology) and visualized by enhanced chemiluminescence detection. Quantifications were performed using ImageJ.

### Tissue preparation and immunofluorescence

As previously described (Bertran-Gonzalez et al., 2008), mice were rapidly anaesthetized by i.p. injection of pentobarbital (500 mg/kg, i.p., Sanofi-Aventis, France) prior to intracardiac perfusion of 4% (weight/vol) paraformaldehyde (PFA) in 0.1 M Na_2_HPO_4_/NaH_2_PO_4_ buffer, pH 7.5, delivered with a peristaltic pump at 20 ml/min during 5 min. Perfusions were performed between ZT 4-10. Thirty-μm thick sections were cut with a vibratome (Leica, France) and processed as follows: free-floating sections were rinsed in Tris-buffered saline (TBS: 0.25 M Tris and 0.5 M NaCl, pH 7.5), incubated for 5 min in TBS containing 3% H_2_O_2_ and 10% methanol and rinsed in TBS. After 15 min incubation in 0.2% Triton X-100 in TBS, sections were rinsed in TBS and incubated overnight at 4°C with the primary antibody: rabbit anti-diphospho signal-regulated kinase (ERK) (phospho-Thr202-Tyr204-ERK1/2, cat-9101; Cell Signaling Technology, Beverly, MA, USA, dilution 1:400) overnight at 4°C. After three rinses in TBS, sections were incubated for 45 min at room temperature with the secondary fluorescent goat antibodies (1:400 Cy3 coupled anti-rabbit IgG, Jackson ImmunoReasearch Europe Ltd) in TBS. Sections were then rinsed twice in TBS and twice in TB and mounted on a slide under coverslips using 1,4-diazabicyclo-[2. 2. 2]-octane (DABCO, Sigma-Aldrich). Confocal microscopy and image analysis were carried out at the Montpellier RIO Imaging Facility. Single-labeled images (636.4 × 636.4 μm) from each region of interest were obtained bilaterally using laser scanning confocal microscopy (Zeiss LSM510 META). Photomicrographs were obtained with the following band-pass setting: Cy3 (band pass filter: 560–615). P-ERK immunoreactive labeled neurons were pseudocolored red. The analysis was performed by counting nuclear or cytosolic Cy3 immunofluorescence. Quantification of immunoreactive cells was performed using Image-J and taking as standard reference a fixed threshold of fluorescence.

### Statistical analysis

Results are presented as mean ± sem. Within group comparisons to chance levels were performed using paired two-tailed *t*-test and differences between groups were analyzed using the unpaired two-tailed *t*-tests or One- or Two-Way analysis of variance (ANOVA) followed by Bonferroni's *post-hoc* test for comparison of multiple groups where applicable. Differences were considered significant for *p* < 0.05. Statistical analyses were performed using GraphPad Prism 5.0 (GraphPad Prism Software Inc., San Diego, USA).

## Results

### Low spontaneous and circadian locomotor activity in *Cry1*^−/−^*Cry2*^−/−^ mice

**Figure 1:**
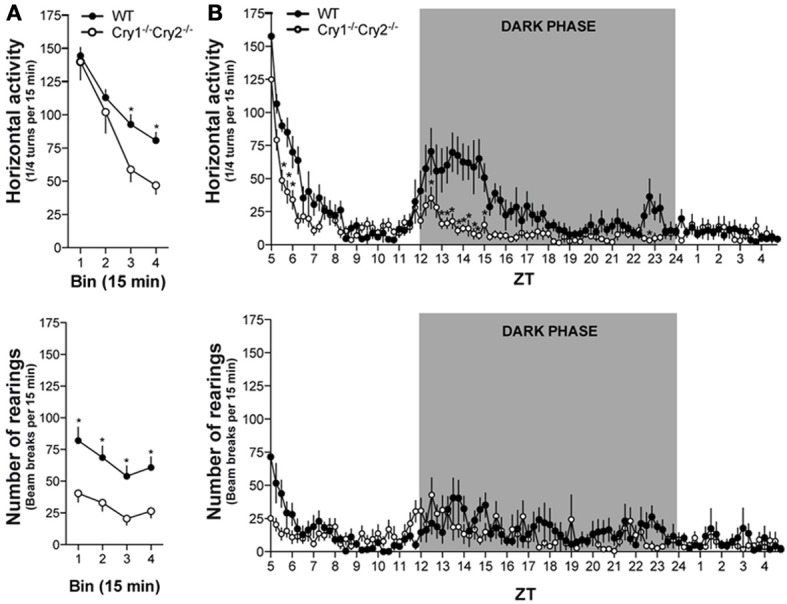
**Spontaneous locomotor activity in *Cry1^−/−^Cry2^−/−^* mice. (A)** When exposed to an enclosed environment, horizontal activity (top panel) declined more rapidly [Genotype × Time: *F*_(3, 111)_ = 3.29, *P* = 0.023; Genotype: *F*_(1, 37)_ = 3.70, *P* = 0.062; Time: *F*_(3, 111)_ = 69.21, *P* < 0.0001] and vertical activity (bottom panel) remained low [Genotype × Time: *F*_(3, 111)_ = 0.27, *P* = 0.84; Genotype: *F*_(1, 37)_ = 14.43, *P* = 0.0005; Time: *F*_(3, 111)_ = 8.66, *P* < 0.0001] during the initial habituation period in *Cry1^−/−^Cry2^−/−^* mice (*n* = 18) compared to wild type (WT) mice (*n* = 21). **(B)** Over a circadian period, the increase in horizontal activity during the dark phase (top panel) was lower [Genotype × Time: *F*_(95, 1520)_ = 3.48, *P* < 0.0001; Genotype: *F*_(1, 16)_ = 49.60, *P* = 0.015; Time: *F*_(95, 1520)_ = 18.54, *P* < 0.0001] in *Cry1^−/−^Cry2^−/−^* mice compared to WT mice. Similarly, the increase in vertical activity during the dark phase (bottom panel) was less pronounced [Genotype × Time: *F*_(95, 1520)_ = 1.59, *P* = 0.0003; Genotype: *F*_(1, 16)_ = 1.79, *P* = 0.20; Time: *F*_(95, 1520)_ = 4.89, *P* < 0.0001] in *Cry1^−/−^Cry2^−/−^* mice (*n* = 10) compared to WT mice (*n* = 8). ^*^P < 0.05 *Cry1^−/−^Cry2^−/−^* mice vs. WT mice.

*Cry1^−/−^Cry2^−/−^* mice are fully arrhythmic in constant darkness but maintain a daily pattern of activity when kept in a 12 h light-dark cycle (van der Horst et al., 1999). We investigated locomotor activity of *Cry1^−/−^Cry2^−/−^* mice in response to novelty, in a non-stressful environment (low luminosity), and over a 24 h period in a 12 h light/dark cycle. When placed in a novel environment, *Cry1^−/−^Cry2^−/−^* mice show an initial period of high horizontal locomotion (ambulations) that declined more rapidly compared to WT mice (Figure 1A, top panel). Moreover, *Cry1^−/−^Cry2^−/−^* mice showed low vertical locomotion (rearings) compared to WT mice throughout the initial habituation period (Figure 1A, bottom panel). Over a 24 h period, WT mice showed a clear daily pattern of activity, with peaks of horizontal locomotion (ambulations) at the onset and at the end of the dark phase. *Cry1^−/−^Cry2^−/−^* mice also had a daily pattern of horizontal activity that increased at the onset of the dark phase but remained low compared to WT mice (Figure 1B, top panel). Similarly, a daily pattern of vertical locomotor activity (rearings) could be observed that peaked at the onset of the dark phase but remained low at the end of the dark phase in *Cry1^−/−^Cry2^−/−^* mice compared to WT mice (Figure 1B, bottom panel). Interestingly, the hypoactive phenotype was specific for mice lacking both *Cry1 and Cry2*. *Cry1^−/−^Cry2^+/+^* and *Cry1^+/+^Cry2^−/−^* single knockout mice showed a normal habituation to a novel environment, and a preserved overall dark phase locomotor activity (Table 1).

**Table 1:** **Behavioral profile of cryptochrome mutant mice**.

		**WT**	**Cry1^−/−^Cry2^+/+^**	**Cry1^+/+^Cry2^−/−^**	**Cry1^−/−^Cry2^−/−^**	
Locomotor activity	Novelty-induced horizontal activity (1/4 turns)	449 ± 26	392 ± 22	433 ± 56	293 ± 29^**^	*F*_(3, 47)_ = 3.89
		(17)	(13)	(11)	**(9)**	*P* = 0.015
	Novelty-induced vertical activity (crosses)	188 ± 35	188 ± 34	164 ± 49	74 ± 16^*^	*F*_(3, 47)_ = 2.34
		(17)	(13)	(11)	**(9)**	*P* = 0.076
	Dark phase horizontal activity (1/4 turns)	1462 ± 160	1218 ± 83	1677 ± 204	476 ± 39^**^	*F*_(3, 47)_ = 9.52
		(17)	(13)	(11)	**(9)**	*P* < 0.0001
	Dark phase vertical activity (crosses)	846 ± 82	907 ± 99	997 ± 174	713 ± 125	*F*_(3, 47)_ = 0.82
		(17)	(13)	(11)	(9)	*P* = 0.49
Forced swim test	Total immobility time (sec)	337 ± 20	336 ± 18	348 ± 24	325 ± 21	*F*_(3, 31)_ = 0.201
		(13)	(9)	(7)	(6)	*P* = 0.89
Open field test	Time in center (%)	137.8 ± 14.3	116.6 ± 18.2	123.0 ± 10.4	40.5 ± 6.4^***^	*F*_(3, 42)_ = 10.68
		(19)	(10)	(10)	**(7)**	*P*< 0.0001
	Number of crosses (number of crosses)	79.0 ± 11.7	55.8 ± 7.2	89.1 ± 6.6	32.33 ± 3.6^*^	*F*_(3, 42)_ = 9.59
		(19)	(10)	(10)	**(7)**	*P*< 0.0001
Elevated plus maze test	Time in open arms (%)	20.9 ± 4.0	5.4 ± 1.5^**^	8.5 ± 5.4^*^	2.5 ± 1.2^***^	*F*_(3, 47)_ = 5.51
		(19)	**(12)**	**(11)**	**(9)**	*P*= 0.001
	Open arm entries (number of entries)	2.8 ± 0.4	0.8 ± 0.2^***^	1.5 ± 0.2^*^	0.8 ± 0.3^***^	*F*_(3, 47)_ = 9.31
		(19)	**(12)**	**(11)**	**(9)**	*P*< 0.0001
	Closed arm entries (number of entries)	10.4 ± 1.0	9.2 ± 1.0	9.0 ± 0.9	9.9 ± 1.2	*F*_(3, 47)_ = 0.40
		(19)	**(12)**	**(11)**	**(9)**	*P*= 0.75
Object recognition test	Time to criterion (sec)	12.4 ± 1.2	7.8 ± 0.6	9.4 ± 1.1	18.7 ± 0.4^***^	*F*_(3, 42)_ = 21.98
		(18)	(11)	(10)	**(7)**	*P*< 0.0001
	Recognition index	0.29 ± 0.04^^**+**++^^	–0.02 ±0.03^***^	–0.01 ± 0.03^***^	–0.08 ± 0.05^***^	*F*_(3, 42)_ = 21.45
		(18)	**(11)**	**(10)**	**(7)**	*P*< 0.0001
Body weight	Aged 10–12 weeks	27.1 ± 0.6	27.9 ± 0.5	28.0 ± 0.5	24.7 ± 0.6^**^	*F*_(3, 54)_ = 21.98
		(19)	(11)	(8)	**(20)**	*P*< 0.0001

* p<0.05,

** p<0.01,

*** p<0.001 vs. WT, One-Way ANOVA and Bonferroni's post-hoc test.

+++ p<0.001 vs chance level one sample t-test.

The number of mice is specified between parentheses.

### Cognitive dysfunction in *Cry1*^−/−^*Cry2*^−/−^ mice

**Figure 2:**
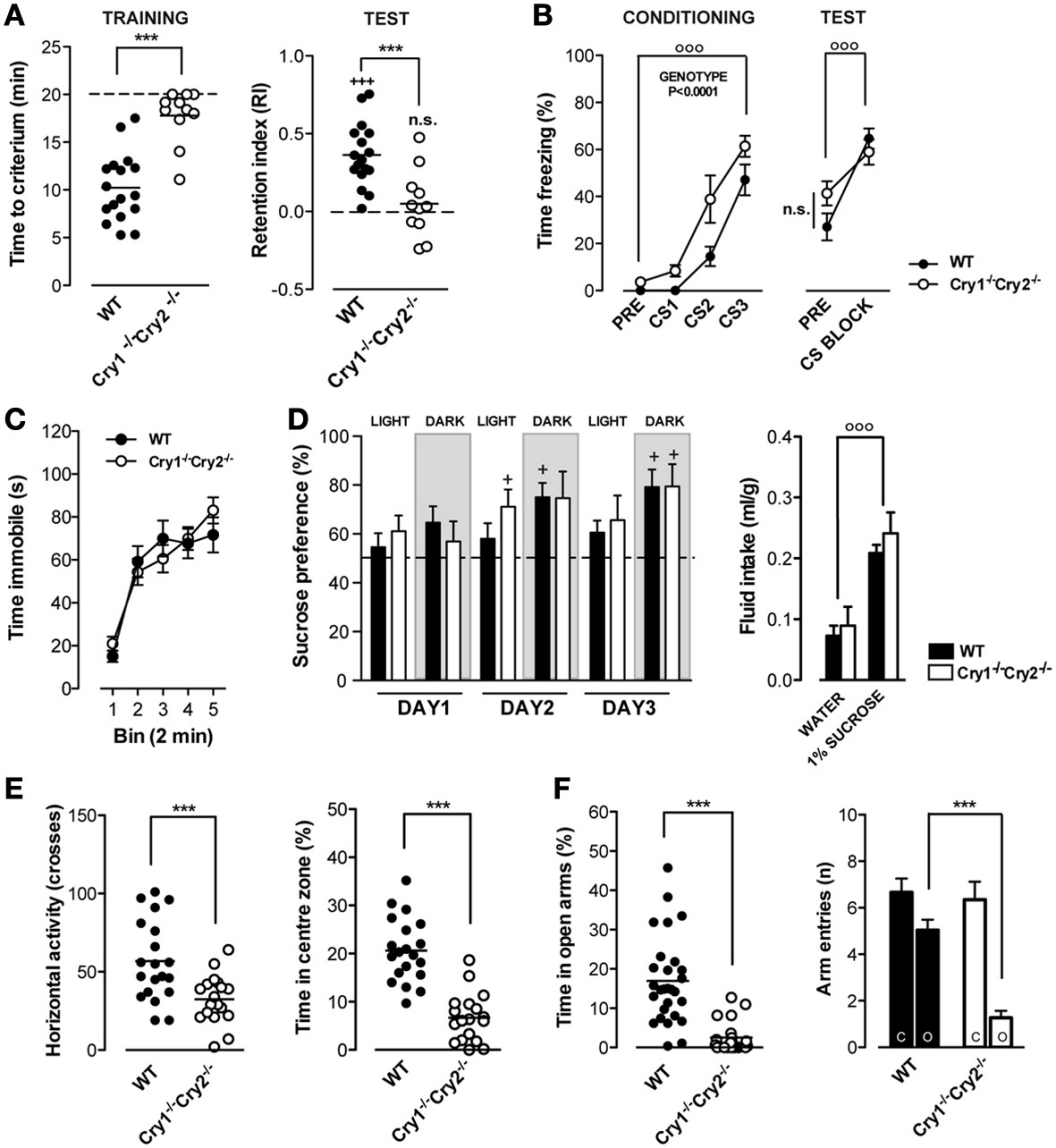
**Cognitive processing, depression-related behavior, and anxiety-related behavior in *Cry1^−/−^Cry2^−/−^* mice. (A)** During the training phase of the object recognition test (left panel), *Cry1^−/−^Cry2^−/−^* mice (*n* = 11) required more time [*t*_(26)_ = 5.971, *P* < 0.0001] to reach the criterion of 40 s total object exploration compared to wild type (WT) mice (*n* = 17). During the test phase, 24 h later, *Cry1^−/−^Cry2^−/−^* mice did not show a preference for the novel object as opposed [*t*_(26)_ = 3.895, *P* = 0.0006] to WT mice, which had a retention index above 0 [*t*_(16)_ = 7.389, *P* < 0.0001]. **(B)** In the auditory fear conditioning test, *Cry1^−/−^Cry2^−/−^* mice (*n* = 8) readily associate the tone with the ensuing foot shock, but display higher [Genotype × Time: *F*_(3, 45)_ = 2.52, *P* = 0.07; Genotype: *F*_(1, 45)_ = 10.81, *P* = 0.005; Time: *F*_(3, 45)_ = 58.98, *P* < 0.0001] freezing levels compared to WT mice (*n* = 10). During the test, 24 h later, both genotypes showed an elevated freezing response to tone presentation [Genotype × Time: *F*_(17, 204)_ = 0.58, *P* = 0.9; Genotype: *F*_(1, 204)_ = 0.03, *P* = 0.86; Time: *F*_(17, 204)_ = 30.32, *P* < 0.0001]. **(C)** In the forced swim test, immobility times did not differ [Genotype × Time: *F*_(4, 96)_ = 1.52, *P* = 0.20; Genotype: *F*_(1, 96)_ = 0.03, *P* = 0.87; Time: *F*_(4, 96)_ = 47.61, *P* < 0.0001] between *Cry1^−/−^Cry2^−/−^* mice (*n* = 8) and WT mice (*n* = 13). **(D)** In the sucrose preference test, both *Cry1^−/−^Cry2^−/−^* mice (*n* = 8) and WT mice (*n* = 12) expressed a clear preference (left panel) for 1% sucrose over water [Genotype × Treatment: *F*_(5, 90)_ = 1.05, *P* = 0.39; Genotype: *F*_(1, 90)_ = 0.12, *P* = 0.73; Treatment: *F*_(5, 90)_ = 5.91, *P* < 0.0001] and total fluid intake (right panel) was significantly higher for 1% sucrose over water in both genotypes [Genotype × Treatment: *F*_(5, 90)_ = 1.05, *P* = 0.39; Genotype: *F*_(1, 90)_ = 0.12, *P* = 0.73; Treatment: *F*_(2, 26)_ = 10.75, *P* < 0.0004]. **(E)** In the open field test, overall activity (left panel) was lower [*t*_(36)_ = 3.498, *P* = 0.0013] in *Cry1^−/−^Cry2^−/−^* mice compared to WT mice. Moreover, the time spent in the center of the open field (right panel) was lower [*t*_(36)_ = 7.225, *P* < 0.0001] in *Cry1^−/−^Cry2^−/−^* mice (*n* = 18) compared to WT mice (*n* = 20). **(F)** In the elevated plus maze test, *Cry1^−/−^Cry2^−/−^* mice (*n* = 18) spent less [*t*_(47)_ = 5.75, *P* < 0.0001] time in the open arms compared to WT mice (*n* = 20). Moreover, the number of arm entries was lower for the open arms but not for the closed arms of the plus maze in *Cry1^−/−^Cry2^−/−^* mice compared to WT mice [Genotype × Arm: *F*_(1, 51)_ = 11.80, *P* = 0.0012; Genotype: *F*_(1, 51)_ = 11.43, *P* = 0.0014; Arm: *F*_(17, 204)_ = 44.67, *P* < 0.0001]. (^***^P < 0.001 *Cry1^−/−^Cry2^−/−^* mice vs. WT mice. ^+^*P* < 0.05 vs. chance level. ^+++^*P* < 0.001 vs. chance level. °°°*P* < 0.001 for treatment or time effect.

Cognitive dysfunction is an endophenotype and co-morbid risk factor for mood, anxiety, and substance abuse disorders (Disner et al., 2011; Millan et al., 2012). We investigated memory formation in cryptochrome mutant mice using the object recognition task. Compared to WT mice, *Cry1^−/−^Cry2^−/−^* mice showed low exploratory drive and required significantly more time to reach the criterion of 40 s total object exploration during the training session (Figure 2A). *Cry1^−/−^Cry2^−/−^* mice that reached this criterion did not show a preference for the novel object during the test session 24 h later as opposed to WT mice (Figure 2A). Interestingly, *Cry1^−/−^Cry2^+/+^* mice and *Cry1^+/+^Cry2^−/−^* mice showed normal object exploration, but were similarly unable to recognize the novel object during the test session 24 h later (Table 1). To ascertain whether the observed learning deficit in *Cry1^−/−^Cry2^−/−^* mice was specific to recognition memory, we further investigated their learning capacity in an auditory fear-conditioning task. *Cry1^−/−^Cry2^−/−^* mice rapidly learned the tone-shock association and displayed significantly higher freezing levels during auditory fear conditioning relative to WT mice. When tested 24 h later, *Cry1^−/−^Cry2^−/−^* mice displayed a clear freezing response to the conditioned tone, which did not differ from WT mice (Figure 2B). Taken together, these data suggest a specific role for both Cry1 and Cry2 genes in non-aversive episodic-like memory formation.

### Lack of spontaneous depression-like behavior in *Cry1*^−/−^*Cry2*^−/−^ mice

Several circadian clock genes have been associated with mood disorders in humans (Soria et al., 2010). This lead us to hypothesize that low exploratory drive in *Cry1^−/−^Cry2^−/−^* mice may be associated with a depression-like phenotype. We investigated the effect of *Cry* gene ablation on depression-like behavior, using a modified version of the forced swim test, with a single swim session to negate potentially confounding memory effects (De Bundel et al., 2013; Gomez-Galan et al., 2013). Surprisingly, we observed that immobility times did not differ between *Cry1^−/−^Cry2^−/−^* and WT mice (Figure 2C). Similarly, immobility times of both *Cry1^−/−^Cry2^+/+^* and *Cry1^+/+^Cry2^−/−^* single knockout mice were not different from those in WT mice (Table 1). In addition, we investigated whether *Cry1^−/−^Cry2^−/−^* showed an altered preference for natural reward. We found no difference in the intake of a 1% sucrose solution between *Cry1^−/−^Cry2^−/−^* and WT mice (Figure 2D). Our data indicate that *cryptochrome* gene deletion does not cause depression-like behavior in mice.

### Increased anxiety-related behavior in *Cry1*^−/−^*Cry2*^−/−^ mice

Circadian clock genes have also been associated with anxiety disorders in humans (Sipila et al., 2010). Given that low exploratory activity and rearing in a novel environment may result from anxiety, we next investigated anxiety-related behaviors in *Cry1^−/−^Cry2^−/−^* mice. Compared to WT mice, *Cry1^−/−^Cry2^−/−^* mice were less active and spent significantly less time exploring the center zone in an open field (Figure 2E). Moreover, *Cry1^−/−^Cry2^−/−^* mice made fewer entries and spent significantly less time in the open arms of an elevated plus maze compared to WT mice (Figure 2F). *Cry1^−/−^Cry2^−/−^* mice made an equal number of closed arm entries indicating increased anxiety in the exposed center of the open field and open arms of the elevated plus maze rather than a general locomotor deficit. Altogether, these data confirmed low exploratory drive in *Cry1^−/−^Cry2^−/−^* mice and revealed that these mutant mice displayed an increased anxiety. Interestingly, open field exploratory activity was not significantly different from WT mice in *Cry1^−/−^Cry2^+/+^* and *Cry1^+/+^Cry2^−/−^* mice (Table 1). However, the number of open arm entries and time spent in the open arms of the elevated plus maze were significantly lower compared to WT mice in *Cry1^−/−^Cry2^+/+^* and in *Cry1^+/+^Cry2^−/−^* mice (Table 1) suggesting that *cryptochromes* influence anxiety-related behaviors.

### Cocaine-induced acute hyperlocomotion and locomotor sensitization in *Cry1*^−/−^*Cry2*^−/−^ mice

**Figure 3:**
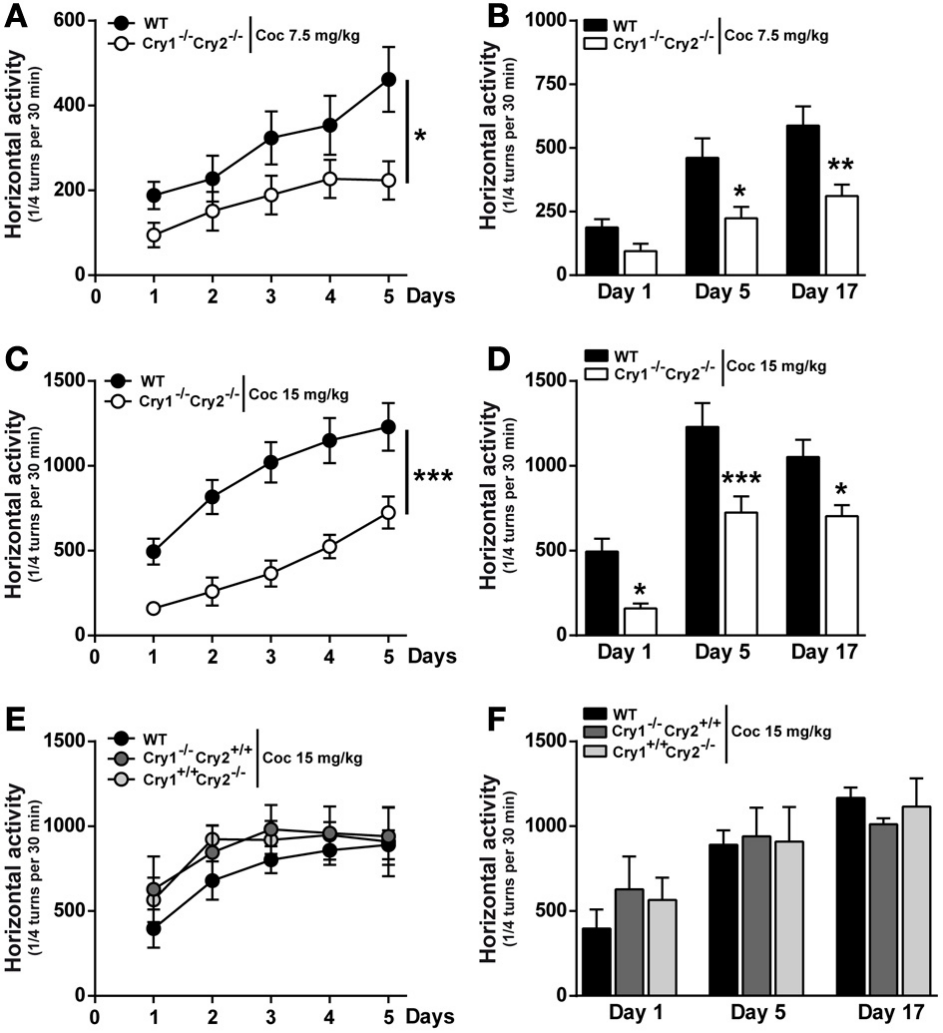
**Cocaine-induced acute hyperlocomotion and locomotor sensitization in *Cry1^−/−^Cry2^−/−^* mice. (A** and **C**) Locomotor activity induced by repeated cocaine administration (7.5 and 15 mg/kg) in wild type (WT) and *Cry1^−/−^Cry2^−/−^* mice (*n* = 10–12 per experimental group). Data (means ± sem) were analyzed using Two-Way ANOVA repeated measures: **A** [Time × Genotype: *F*_(4, 76)_ = 2.783, *P* = 0.0325; Time: *F*_(4, 76)_ = 17.95, *P* < 0.0001; Genotype: *F*_(1, 19)_ = 3.883, *P* = 0.0635], **C** [Time × Genotype: *F*_(4, 76)_ = 3.302, *P* = 0.015; Time: *F*_(4, 76)_ = 53.45, *P* < 0.0001; Genotype: *F*_(1, 19)_ = 17.98, *P* = 0.0004]. **(B** and **D**) Cumulative locomotor activity of WT and *Cry1^−/−^Cry2^−/−^* mice over a 30 min period post cocaine administration. Data (means ± sem) were analyzed using Two-Way ANOVA repeated measures: **(B)** [Time × Genotype: *F*_(2, 38)_ = 3.907, *P* = 0.0288; Time: *F*_(2, 38)_ = 41.05, *P* < 0.0001; Genotype: *F*_(1, 19)_ = 8.998, *P* = 0.0074], **(D)** [Time × Genotype: *F*_(2, 38)_ = 0.8807, *P* = 0.4228; Time: *F*_(2, 38)_ = 48.61, *P* < 0.0001; Genotype: *F*_(1, 19)_ = 14.73, *P* = 0.0012]. **(E)** Locomotor activity induced by repeated cocaine administration (15 mg/kg) in WT (*n* = 12), *Cry1^−/−^/Cry2^+/+^* (*n* = 12) and *Cry1^+/+^/Cry2^−/−^* (*n* = 5) mice. Data (means ± sem) were analyzed using Two-Way ANOVA repeated measures: [Time × Genotype: *F*_(8, 104)_ = 0.3432, *P* = 0.9470; Time: *F*_(4, 104)_ = 9.940, *P* < 0.0001; Genotype: *F*_(2, 26)_ = 0.4848, *P* = 0.6213]. **(F)** Cumulative locomotor activity of WT, *Cry1^−/−^/Cry2^+/+^* and *Cry1^+/+^/Cry2^−/−^* mice over a 30 min period post cocaine Data (means ± sem) were analyzed using Two-Way ANOVA repeated measures: [Time × Genotype: *F*_(4, 52)_ = 0.9167, *P* = 0.4613; Time: *F*_(2, 52)_ = 16.52, *P* < 0.0001; Genotype: *F*_(2, 26)_ = 0.07071, *P* = 0.9319]. ^*^p < 0.05, ^**^p < 0.01, ^***^p < 0.001.

Recent studies highlight a link between disrupted circadian rhythms and cocaine sensitivity (Falcon and McClung, 2009). Single administration of cocaine induces acute locomotor hyperactivity and repeated administrations induce locomotor sensitization or incremental locomotor hyperactivity in rodents (Brami-Cherrier et al., 2005). As illustrated in Figure 3, single administration of cocaine (Day1, 7.5 and 15 mg/kg) induced higher locomotor responses in WT mice compared to *Cry1^−/−^Cry2^−/−^* mice (Figures 3A–D). When normalizing locomotor activity following cocaine injection to baseline activity, cocaine-induced hyperlocomotion remained significantly higher in WT mice compared to *Cry1^−/−^Cry2^−/−^* mice (Supplemental Figure 1). Despite this reduced acute hyperlocomotion, *Cry1^−/−^Cry2^−/−^* mice develop locomotor sensitization following repeated administration of cocaine (7.5 and 15 mg/kg) (Figures 3A–D). On the other hand, *Cry1^−/−^Cry2^+/+^* and *Cry1^+/+^Cry2^−/−^* mice displayed a normal acute and sensitized locomotor response to cocaine (Figures 3E,F). These results suggest that *Cry* genes are involved in the acute locomotor effect induced by cocaine but are not required for sensitization.

### Cocaine-induced ERK activation in *Cry1*^−/−^*Cry2*^−/−^ mice

**Figure 4:**
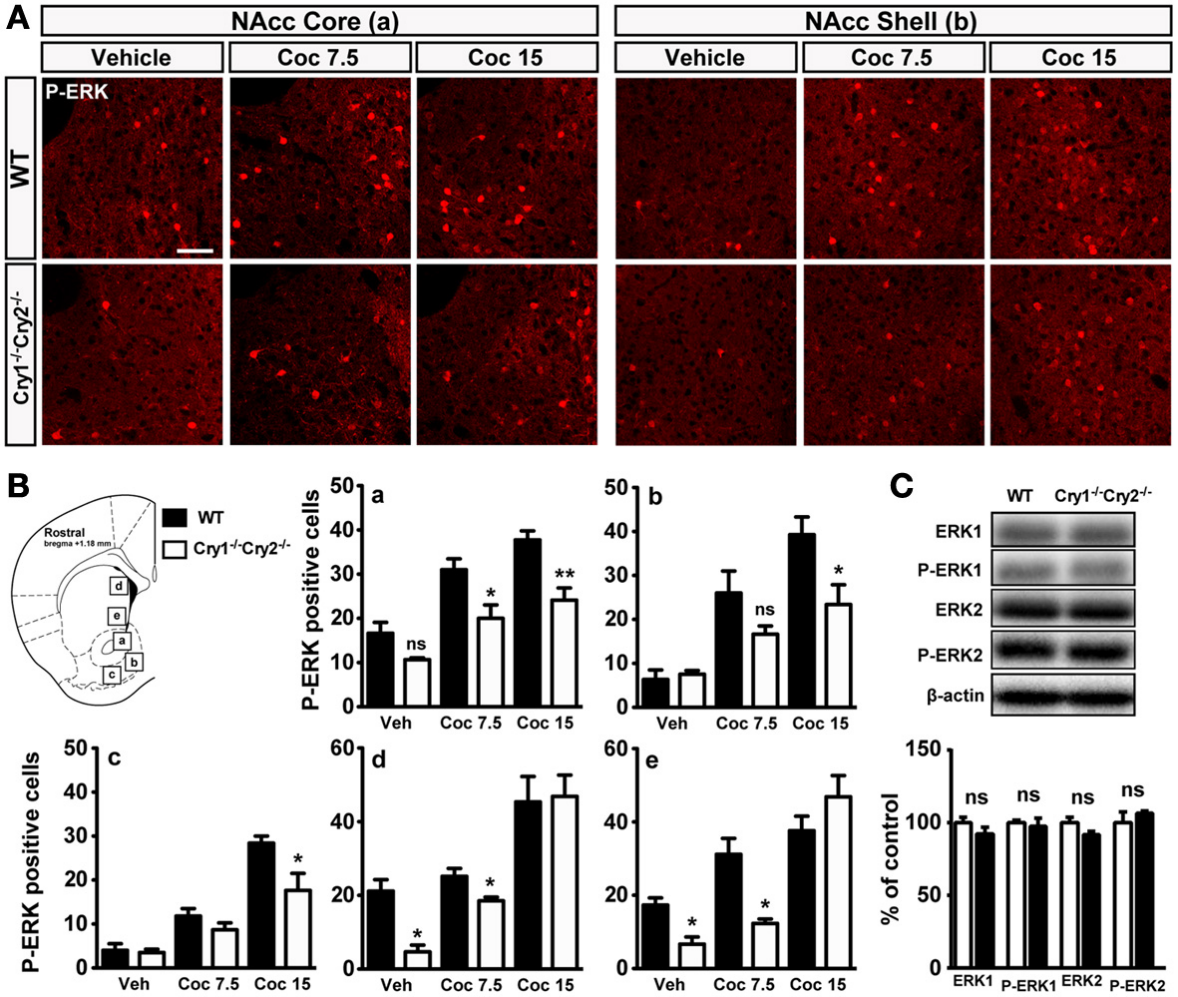
**Cocaine-induced ERK activation in *Cry1^−/−^Cry2^−/−^* mice. (A)** Immunofluorescence pictures showing P-ERK in the NAcc Core and Shell of wild type (WT) and *Cry1^−/−^Cry2^−/−^* mice treated with vehicle (veh) (WT, *n* = 3; *Cry1^−/−^Cry2^−/−^*, *n* = 3), cocaine 7.5 mg/kg (Coc7.5) (WT, *n* = 3; *Cry1^−/−^Cry2^−/−^*, *n* = 3) and cocaine 15 mg/kg (Coc15) (WT, *n* = 4; *Cry1^−/−^Cry2^−/−^*, *n* = 4). **(B)** Quantification of P-ERK positive neurons in NAcc core (a), NAcc medial shell (b), NAcc ventral shell (c), dorso-medial striatum (d), and ventro-medial striatum (e). Data (means ± sem) were analyzed using an unpaired Student's *t*-test: ^*^p < 0.05, ^**^p < 0.01. **(C)** Quantification of ERK1 and ERK2 expression levels in wild type (WT) (*n* = 6) and *Cry1^−/−^Cry2^−/−^* mice (*n* = 6) (means ± sem).

Acute administration of drugs of abuse, including cocaine, leads to a rapid and transient activation of ERK in the dorsal striatum and in the nucleus accumbens (NAcc) (Valjent et al., 2000, 2004; Gangarossa et al., 2013). As such, ERK can be used as a marker for neuronal activity. Moreover, ERK activation participates in some long-lasting behavioral changes induced by cocaine (Lu et al., 2006; Girault et al., 2007). To evaluate possible changes in striatal ERK activity, we examined the levels of ERK phosphorylation by immunofluorescence 10 min after a single cocaine administration (7.5 or 15 mg/kg). Consistent with previous findings (Bertran-Gonzalez et al., 2008), cocaine (7.5 and 15 mg/kg) elicited a significant increase in the number of P-ERK positive cells within the dorsal striatum and the NAcc core and shell of WT mice (Figures 4A,B). Although, basal ERK phosphorylation is strongly reduced in the dorsal striatum of *Cry1^−/−^Cry2^−/−^* mice, this effect was significantly diminished within the dorsal striatum and the NAcc core and displayed a trend toward a decrease in the NAcc shell when *Cry1^−/−^Cry2^−/−^* mice were injected with 7.5 mg/kg of cocaine (Figures 4A,B). The opposite profile was observed when cocaine at the dose of 15 mg/kg was administrated (Figures 4A,B). Importantly, the observed decrease in ERK phosphorylation is not a consequence of a low total level of ERK since western blot analysis of striatal total protein ERK did not reveal a significant difference in *Cry1^−/−^Cry2^−/−^* mice (Figure 4C). It is interesting to note that the slight decrease observed on basal ERK phosphorylation apparent by immunofluorescence was not detected by immunoblotting. This discrepancy may be attributable to the fact that immunoblotting detects an average of signaling events occurring in mixed populations of cells while immunofluorescence allows the identification of specific populations of cells in which a strong activation of ERK takes place. Overall, these data indicate that basal ERK phosphorylation is lower in medium-sized spiny neurons in *Cry1^−/−^Cry2^−/−^* mice compared to WT mice but that the cocaine is nevertheless able to elicit ERK activation. These data correlate with the low baseline activity in *Cry1^−/−^Cry2^−/−^* mice but the preserved sensitization to cocaine, which depends on ERK phosphorylation in the striatum (Valjent et al., 2000).

## Discussion

In the present study, we characterized the behavioral consequences of full genetic disruption of the circadian clock, through deletion of both paralogs of the core circadian transcriptional repressor *cryptochrome* (*Cry1* and *Cry2*). Our findings revealed that the lack of *Cry1* and *Cry2 genes* (*Cry1^−/−^Cry2^−/−^*) elicits cognitive dysfunction, promotes anxiety-related behavior and results in a decreased sensitivity to cocaine but does not affect depression-like behavior. Interestingly, cognitive function and anxiety-related behaviors but none of the other behavioral parameters were also altered in mice lacking either *Cry1* or *Cry2*.

We found that *Cry1*^−/−^/*Cry2*^−/−^ mice maintain a daily pattern of activity when kept in a normal light-dark cycle but are hypoactive during the dark phase. Moreover, *Cry1*^−/−^/*Cry2*^−/−^ mice showed a rapid decline in activity following exposure to a novel environment compared to WT mice, supporting the notion that circadian clock proteins may control adaptation to novelty (Kondratova et al., 2010). No difference in locomotor activity was observed between WT mice and single knockout mice for *Cry1* or *Cry2*. Interestingly, all *cryptochrome* mutants showed lower overall performance compared to WT in the rotarod task, but motor performance improved in all strains, indicating that Cry1 and Cry2 genes are not required for motor learning (Supplemental Figure 2). Low initial rotarod performance in *cryptochrome* mutants may reflect elevated anxiety in this task. Similar to our observations in single *cryptochrome* mutants, no overt differences in spontaneous locomotor activity were previously described in single knockout mice for the circadian transcriptional repressors *mPer1* or *mPer2* (Abarca et al., 2002; Spencer et al., 2013). In contrast, knockout mice for the circadian transcriptional activators *Clock*, *Bmal1* and *NPAS2* are hyperactive (Dudley et al., 2003; McClung, 2006; Kondratova et al., 2010). These results support the notion that different circadian clock genes can mediate opposing effects on locomotor behavior, corresponding to their role as transcriptional activators or repressors.

The circadian system has been proposed to play a critical role in memory formation (Gerstner and Yin, 2010; Kondratova et al., 2010). Several pathways implicated in memory formation show circadian oscillation (Eckel-Mahan and Storm, 2009). However, the precise involvement of circadian clock genes and their role as core pacemakers on memory formation is less clear. Mice lacking *NPAS2* show impaired cued and contextual fear learning (Garcia et al., 2000). Similarly, BMAL1 knockout mice show impaired contextual habituation (Kondratova et al., 2010). In contrast to these mutants for circadian transcriptional activators, mice lacking *mPer1* and *mPer2* show normal spatial learning and contextual fear learning (Zueger et al., 2006; Feillet et al., 2008). Similarly, knockout mice for both *Cry1* and *Cry2* display normal spatial learning (Van der Zee et al., 2008) and intact cued (present study) and contextual fear learning (Van der Zee et al., 2008). Interestingly, *Cry1*^−/−^/*Cry2*^−/−^ mice have intact short-term interval timing (Papachristos et al., 2011) but are unable to learn place-time-of-day associations (Van der Zee et al., 2008). Our study clearly shows learning deficits in the object recognition task in *Cry* double knockout mice as well as *Cry1* and *Cry2* single knockout mice. Memory impairments in *Cry* mutants are unlikely to result from low attention given that *Cry1* and *Cry2* mutant mice showed normal object exploration. One potential explanation for the specificity of these cognitive deficits is that Cry mutants may display learning deficits when cognitive demands are higher (place vs. place-time-of-day associations) or when the incentive for learning is lower (spontaneous exploration vs. food reward or shock punishment). Moreover, these data indicate that different circadian clock genes disruption affects specific types of learning.

Aberrant light-dark rhythms directly induce depression-like behavior in rodents (Gonzalez and Aston-Jones, 2008; Fonken et al., 2009; Monje et al., 2011). This has led to the hypothesis that alterations in light may indirectly alter mood through changes in the circadian clock. However, a recent study demonstrated that mice exposed to a 3.5 h-light/3.5 h-dark cycle developed depression-like behavior while maintaining a normal amount of sleep and without showing significant disruption of circadian clock components (LeGates et al., 2012). Conversely, we here show that full disruption of the circadian clock through deletion of the paralog pair of *cryptochromes* did not cause depression-related behaviors in mice maintained in a standard 12 h-light/12 h-dark cycle. This further suggests that altered mood in other circadian clock gene mutants may be a consequence of the role of clock gene products as transcription factors and altered expression of their tissue-specific target genes. *Clock* mutant mice display reduced depression-like behaviors (Easton et al., 2003; Roybal et al., 2007). Interestingly, *Per2^Brdm1^* mutant mice that show lower activity and expression of MAO-A and display a depression-resistant-like phenotype (Hampp et al., 2008). These effects may depend on paralog-specific gene interactions. For instance, the E-box element of the MAO-A promoter was shown to serve as a binding site for the NPAS2/BMAL1 heterodimer but not for the CLOCK/BMAL1 heterodimer (Hampp et al., 2008). In the same study, m*Per2* strongly increased, whereas *Cry1* only marginally decreased transcriptional regulation of MAO-A (Hampp et al., 2008). We found no significant effect of *Cry1* and/or *Cry2* gene inactivation on MAO-A expression (data not shown) or depression-like behavior in the forced swim test. Moreover, *Afterhours* mutant mice, carrying an FBXL3 mutation resulting in decreased ubiquitination and degradation of CRY (Busino et al., 2007; Godinho et al., 2007; Siepka et al., 2007), showed no spontaneous depression-like behavior in the forced swim test (Keers et al., 2012). These findings further indicate that cryptochromes may not have a critical influence on depression-related behavior and raises the question of causality for described associations between *Cry1* and *Cry2* single nucleotide polymorphisms with mood disorders in humans (Lavebratt et al., 2010; Soria et al., 2010).

Few studies have investigated the effect of circadian clock genes on anxiety. Whereas clock mutant mice appear to be less anxious (Easton et al., 2003; Roybal et al., 2007), mice lacking both *mPer1* and *mPer2* showed elevated anxiety in a range of behavioral tests, whereas no phenotypes were observed in single deficient mice (Spencer et al., 2013). *Cry1* or *Cry2* single knockout mice showed elevated anxiety in the elevated plus maze, but less prominent compared to mice lacking both *Cry1* and *Cry2* that displayed robust anxiety-related behaviors across tests. This is in line with the observation that *Afterhours* mutant mice showed lower anxiety-related behaviors (Keers et al., 2012) and suggests largely complementary roles of Cry1 and Cry2 with an opposite influence of the circadian transcriptional activators and repressors on anxiety-related behavior. Lesions of the suprachiasmatic nucleus elicit antidepressant-like effects but do not affect anxiety-related behaviors (Engelmann et al., 1998; Tataroglu et al., 2004; Tuma et al., 2005). This suggests that elevated anxiety in *mPer1^−/−^mPer2^−/−^* mice and in *Cry1^−/−^Cry2^−/−^* mice may result from lack of function of these circadian clock genes in brain regions outside of the suprachiasmatic nucleus, such as the nucleus accumbens (Spencer et al., 2013).

Finally, circadian clock genes have been implicated in susceptibility to substance abuse disorders. Thus, ClockΔ 19 mutant mice are more sensitive to the psychostimulant and rewarding effects of cocaine (McClung et al., 2005; Roybal et al., 2007) and self-administer more cocaine than WT mice (Ozburn et al., 2012). Although, *mPer1^−/−^* mice are hyper responsive to the acute effects of cocaine and show a sensitized response following a single administration (Abarca et al., 2002), cocaine-induced self-administration and reinstatement do not differ from WT mice (Halbout et al., 2011). Interestingly, *mPer2^−/−^* mice show a radically different phenotype (Abarca et al., 2002). While the acute response is decreased, *mPer2^−/−^* mice display an exacerbated sensitization to the locomotor effects of cocaine (Abarca et al., 2002). Our study reveals that *Cry1* and *Cry2* single knockout mice show normal acute and sensitized responses to cocaine. *Cry1^−/−^Cry2^−/−^* double knockout mice display reduced acute cocaine-induced hyperlocomotion, but they still develop locomotor sensitization to repeated administrations of cocaine. Similarly, baseline ERK phosphorylation was overall lower in *Cry1^−/−^Cry2^−/−^* double knockout mice, but ERK phosphorylation increased following cocaine administration in the dorsal striatum and the NAcc, possibly accounting for the development of locomotor sensitization (Corvol et al., 2007). Altogether, our data suggest that in contrast to the other circadian clock genes, cryptochromes may not have a critical influence on cocaine-related behavior.

In conclusion, this study shows that disruption of the circadian clock, through deletion of one and/or both paralogs of the circadian core gene *cryptochrome* elicits cognitive dysfunction and promotes anxiety-related behaviors. In contrast, depression-related behaviors seem to be unaffected in *cryptochrome* mutant lines, and cocaine-related behaviors were only affected in mice lacking both *cryptochrome* paralogs during the acute phase of drug administration. Future studies will be necessary to decipher the mechanisms underlying the observed associations.

### Conflict of interest statement

The authors declare that the research was conducted in the absence of any commercial or financial relationships that could be construed as a potential conflict of interest.

## Supplementary material

Figure S1 **Cocaine-induced acute hyperlocomotion**. Locomotor activity induced by acute cocaine administration (7.5 and 15 mg/kg) in wild type (WT) and *Cry1*^−/−^*Cry2*^−/−^ mice (*n* = 10–12 per experimental group). Measurements are shown as **(A)** raw data and **(B)** normalized data on the last day of habituation. Data (means ± sem) were analyzed using an unpaired Student's *t*-test: ^*^*p* < 0.05, ^**^*p* < 0.01.

Figure S2 **Motor coordination and learning in *Cry1^−/−^Cry2^−/−^* mice. (A)** Curve showing coordination and motor learning in WT (*n* = 16), *Cry1^−/−^Cry2^+/+^* (*n* = 5), *Cry1^+/+^Cry2^−/−^* (*n* = 6) and *Cry1^−/−^Cry2^−/−^* (*n* = 8) mice over a training period of 3 days. Data (means ± sem) were analyzed using Two-Way ANOVA repeated measures [Time × Genotype: *F*_(6, 62)_ = 0.1692, *P* = 0.7813; Time: *F*_(2, 62)_ = 57.44, *P* < 0.0001; Genotype: *F*_(3, 31)_ = 22.40, *P* < 0.0001]. (^***^*p* < 0.001 mutant mice vs. WT mice). **(B)** Percentage of learning in WT (*n* = 16), *Cry1^−/−^Cry2^+/+^* (*n* = 5), *Cry1^+/+^Cry2^−/−^* (*n* = 6) and *Cry1^−/−^Cry2^−/−^* (*n* = 8) Data (means ± sem) were analyzed using Two-Way ANOVA repeated measures [Time × Genotype: *F*_(3, 31)_ = 2.034, *P* = 0.1294; Time: *F*_(1, 31)_ = 113.8, *P* < 0.0001; Genotype: *F*_(3, 31)_ = 0.9203, *P* = 0.4425]. (^***^*p* < 0.001 Day 1 vs. Day 3).
