# Supplementary figures and images for: Cognitive dysfunction, elevated anxiety, and reduced cocaine response in circadian clock-deficient cryptochrome knockout mice
Source: Front Behav Neurosci. 2013 Oct 24;7:152. doi: 10.3389/fnbeh.2013.00152 (PMC3807562; doi:10.3389/fnbeh.2013.00152)

# Supplemental Figure 1

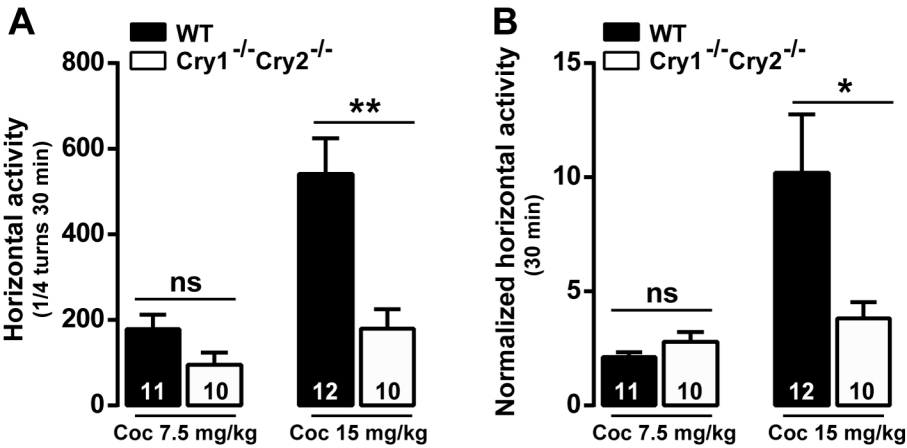

Supplement: Figure S1 — Cocaine-induced acute hyperlocomotion. Locomotor activity induced by acute cocaine administration (7.5 and 15 mg/kg) in wild type (WT) and Cry1−/−Cry2−/− mice (n = 10–12 per experimental group). Measurements are shown as (A) raw data and (B) normalized data on the last day of habituation. Data (means ± sem) were analyzed using an unpaired Student's t-test: *p < 0.05, **p < 0.01. [file Presentation1.PDF]

# Supplemental Figure 2

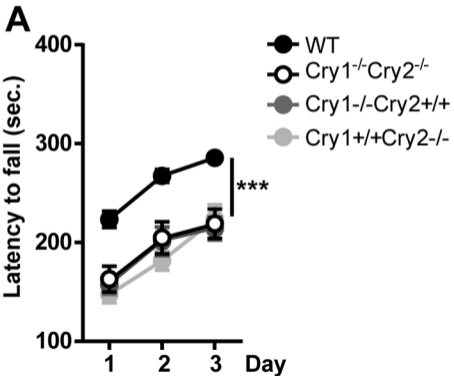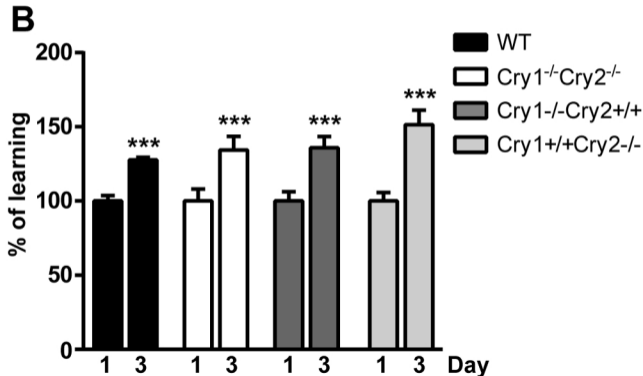

Supplement: Figure S2 — Motor coordination and learning in Cry1−/−Cry2−/− mice. (A) Curve showing coordination and motor learning in WT (n = 16), Cry1−/−Cry2+/+ (n = 5), Cry1+/+Cry2−/− (n = 6) and Cry1−/−Cry2−/− (n = 8) mice over a training period of 3 days. Data (means ± sem) were analyzed using Two-Way ANOVA repeated measures [Time × Genotype: F(6, 62) = 0.1692, P = 0.7813; Time: F(2, 62) = 57.44, P < 0.0001; Genotype: F(3, 31) = 22.40, P < 0.0001]. (***p < 0.001 mutant mice vs. WT mice). (B) Percentage of learning in WT (n = 16), Cry1−/−Cry2+/+ (n = 5), Cry1+/+Cry2−/− (n = 6) and Cry1−/−Cry2−/− (n = 8) Data (means ± sem) were analyzed using Two-Way ANOVA repeated measures [Time × Genotype: F(3, 31) = 2.034, P = 0.1294; Time: F(1, 31) = 113.8, P < 0.0001; Genotype: F(3, 31) = 0.9203, P = 0.4425]. (***p < 0.001 Day 1 vs. Day 3). [file Presentation2.PDF]
